# Immune Challenges and Seizures: How Do Early Life Insults Influence Epileptogenesis?

**DOI:** 10.3389/fphar.2020.00002

**Published:** 2020-02-04

**Authors:** Bridgette D. Semple, Larissa K. Dill, Terence J. O'Brien

**Affiliations:** ^1^ Department of Neuroscience, Central Clinical School, Monash University, Melbourne, VIC, Australia; ^2^ Department of Medicine, Royal Melbourne Hospital, The University of Melbourne, Parkville, VIC, Australia; ^3^ Department of Neurology, Alfred Health, Melbourne, VIC, Australia

**Keywords:** epilepsy, seizure, immune response, cytokines, interleukin-1, brain injury, neuroinflammation, development

## Abstract

The development of epilepsy, a process known as epileptogenesis, often occurs later in life following a prenatal or early postnatal insult such as cerebral ischemia, stroke, brain trauma, or infection. These insults share common pathophysiological pathways involving innate immune activation including neuroinflammation, which is proposed to play a critical role in epileptogenesis. This review provides a comprehensive overview of the latest preclinical evidence demonstrating that early life immune challenges influence neuronal hyperexcitability and predispose an individual to later life epilepsy. Here, we consider the range of brain insults that may promote the onset of chronic recurrent spontaneous seizures at adulthood, spanning intrauterine insults (e.g. maternal immune activation), perinatal injuries (e.g. hypoxic–ischemic injury, perinatal stroke), and insults sustained during early postnatal life—such as fever-induced febrile seizures, traumatic brain injuries, infections, and environmental stressors. Importantly, all of these insults represent, to some extent, an immune challenge, triggering innate immune activation and implicating both central and systemic inflammation as drivers of epileptogenesis. Increasing evidence suggests that pro-inflammatory cytokines such as interleukin-1 and subsequent signaling pathways are important mediators of seizure onset and recurrence, as well as neuronal network plasticity changes in this context. Our current understanding of how early life immune challenges prime microglia and astrocytes will be explored, as well as how developmental age is a critical determinant of seizure susceptibility. Finally, we will consider the paradoxical phenomenon of preconditioning, whereby these same insults may conversely provide neuroprotection. Together, an improved appreciation of the neuroinflammatory mechanisms underlying the long-term epilepsy risk following early life insults may provide insight into opportunities to develop novel immunological anti-epileptogenic therapeutic strategies.

## Introduction

Epilepsy may develop later in life following a prenatal or early postnatal insult such as cerebral ischemia, stroke, brain trauma, or infection. These so-called “acquired epilepsies” account for approximately one-third of all human epilepsies (Engel, 1996; Thomas and Berkovic, 2014), and present clinically after a latent period of variable length (months to years) following the precipitating insult. During this time the brain undergoes progressive changes in neuronal connectivity and intrinsic excitability to ultimately result in an increased propensity for spontaneous recurrent seizures (a process known as “epileptogenesis”) to occur (Lowenstein, 1996; Herman, 2002). While diverse in nature, early life insults that have been associated with the subsequent development of epilepsy share common pathophysiological pathways involving innate immune activation, including neuroinflammation, which is proposed to play a critical role in epileptogenesis (Vezzani et al., 2011a; Becker, 2018).

The developing brain undergoes significant dynamic changes during fetal and early postnatal life, rendering it particularly vulnerable to insults and stressors which can have either transient or permanent effects on neuronal function (Herlenius and Lagercrantz, 2004). Indeed, the developing brain at baseline has an increased propensity for seizure activity compared to the adult brain (Hauser, 1995), thought to be attributed at least in part to the abundant excitatory circuits but fewer inhibitory circuits in the neonatal brain (Nardou et al., 2013). Further, the developing brain appears to be primed to respond to immune challenges in such a way that predisposes the brain towards seizure induction (Bilbo and Schwarz, 2012; Nasr et al., 2019). Immune challenges and the subsequent immune response, including neuroinflammation, are increasingly recognized as an important factor in the pathophysiology of seizure generation, seizure-related neuropathology, and epileptogenesis (Galic et al., 2008; Riazi et al., 2010; Vezzani et al., 2011a).

Several mechanisms have been proposed to explain how and why prenatal, perinatal, and postnatal insults, such as those described above, result in a vulnerability to develop acquired epilepsy later in life. For example, modulation of gene transcription and epigenetic programming, acquired channel and synaptopathies, and neuronal network connectivity all likely play an important role, as discussed elsewhere (Becker, 2018). Here, we focus on evidence surrounding the hypothesis that inflammation promotes epileptogenesis, elaborating on data regarding soluble inflammatory mediators as well as cellular contributions in this process.

Neuroinflammation, defined as an inflammatory response within the brain or spinal cord, is a common consequence of brain injuries, insults, and immune challenges (Disabato et al., 2017). Characterized by the release of inflammatory mediators including pro- and anti-inflammatory cytokines, complement proteins and danger signals, activation of innate immune cells, astrocytes and microglia, and recruitment of blood-derived leukocytes into the central nervous system (CNS), neuroinflammation is also a common feature of temporal lobe epilepsy (TLE) in both patients and animal models (Ravizza et al., 2011; Vezzani et al., 2011a; De Vries et al., 2016). As reviewed in depth elsewhere, increasing evidence suggests that inflammation represents a causal mechanism that can also initiate and perpetuate seizure activity (Vezzani et al., 2011a; Webster et al., 2017), contributing to both ictogenesis (the onset of a seizure) and epileptogenesis (Vezzani et al., 2019).

**Figure 1:**
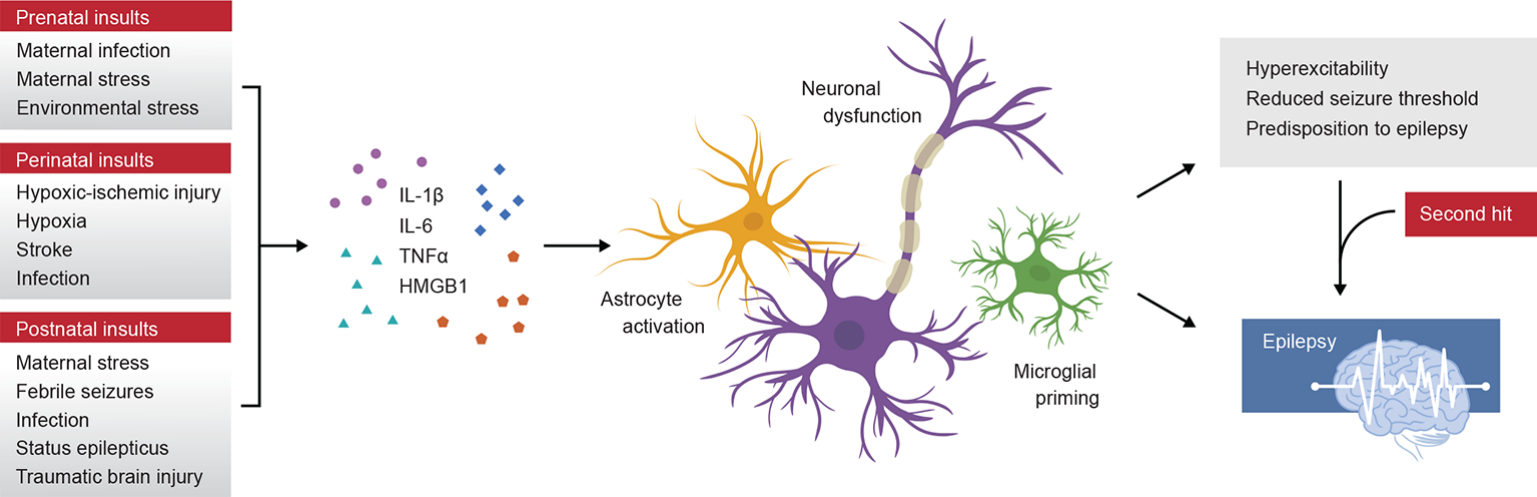
Schematic summary of prenatal, perinatal, and postnatal insults to the developing human brain that initiate an inflammatory immune response, including the release of pro-inflammatory cytokines interleukin (IL)-1β, tumor necrosis factor alpha (TNFα), IL-6 and others. Experimental models have revealed that these cytokines promote astrocyte and microglial reactivity, and contribute to neuronal dysfunction by several mechanisms including alterations in neurotransmitter receptor subunit expression. These changes may lead to hyperexcitability or a reduced seizure threshold, resulting in an increased vulnerability to epilepsy. Epilepsy may develop over time and can be accelerated or triggered by a second-hit insult, such as a later life immune challenge.

In this review, we will consider the most common early life insults linked to the development of epilepsy later in life—including prenatal immune activation, perinatal injuries, and immune challenges sustained during early postnatal life (such as infections, neurotrauma, and even seizures themselves) (**Figure 1**). While not all of the described insults are purely immune-mediated—and indeed, are known to involve other biological mechanisms (e.g. environmental stress and neurotrauma)— we have incorporated these insults here in review to highlight how a range of distinctly different insults during early life can similarly yield a propensity for later life epilepsy. Much of the mechanistic evidence to date is preclinical, utilizing rodent models at postnatal day (p) 0–5 to correspond roughly to the third trimester prenatal in humans, p7–12 equivalent to the human infant at birth, and p21 to model the transition to early childhood (Semple et al., 2013). Specifically, we focus on evidence of the neurobiological mechanisms underlying the chronic consequences of such insults; in particular, the neuroinflammatory response. Together, this review provides a wide-ranging overview of how and why epilepsy may develop after insults to the developing brain *via* neuroimmune modulation. Such an understanding is necessary to inform the development and appropriate application of novel therapeutic agents targeting the relevant biological mechanisms, with the goal of disrupting and preventing the epileptogenic process from occurring.

## Prenatal Insults

Prenatal life is a time of unique immunological status for a developing fetus, which is intricately associated with maternal health status. A large and growing body of literature provides evidence that infections and other immune challenges sustained during pregnancy can influence fetal brain development, with *in utero* exposure to infections and/or inflammation considered to be an environmental risk factor for neurodevelopmental and psychiatric disorders including autism and schizophrenia (Solek et al., 2018; Guma et al., 2019).

Epidemiological data has suggested a relationship between maternal infections and a high incidence of childhood epilepsy in offspring (Norgaard et al., 2012). Several large population-based cohort studies have reported the greatest risk of epilepsy in the offspring of mothers who sustained infections resulting in fever during early to mid-pregnancy (Sun et al., 2008; Sun et al., 2011). Experimentally, this scenario can be modeled in rodents by evoking an infection-like immune challenge to pregnant dams, then assessing the seizure susceptibility of the resulting offspring. Lipopolysaccharide (LPS), a component of the cell wall of gram-negative bacteria and commonly used experimental immunogen to model a bacterial infection, results in persistent changes in neuronal excitability *in vitro* (Gullo et al., 2014), and exacerbates hippocampal excitability in electrical kindling models *in vivo* (Auvin et al., 2010b). When embryos are exposed to LPS *via* inoculation of the pregnant dam at gestational days 15–16, a second challenge at p21—injection of the L-glutamate analog kainic acid (KA)—revealed increased seizure susceptibility compared to those exposed to saline control (Yin et al., 2015). This finding was associated with exacerbated, long-lasting astrogliosis, and worsened spatial learning ability when assessed at adulthood (Yin et al., 2015). Astrocytes, as the most numerous glial cells in the CNS, play many essential roles in tissue homeostasis, synaptic transmission, and neuroimmune responses (Farina et al., 2007; Clarke and Barres, 2013). Accumulating compelling evidence suggests that aberrant astrocyte activation contributes to the pathophysiology of epilepsy (De Lanerolle et al., 2010; Yin et al., 2015; Patel et al., 2019). Together with epidemiological evidence that systemic inflammation increases an individuals' susceptibility to seizures by lowering their seizure threshold (Yuen et al., 2018), these studies provide the foundation for the hypothesis that inflammation is a critical modulator of brain excitability.

Polyinosinic:polycytidylic acid (poly I:C) is an experimental substrate frequently used to mimic viral infections. When administered to gestating animals in a model known as maternal immune activation (MIA), this toll-like receptor 3 (TLR3) agonist results in long-lasting physiological perturbations (Meyer, 2014). Poly I:C administration to pregnant mice between embryonic days 12 to 16 results in the offspring exhibiting increased vulnerability to hippocampal kindling, with strong evidence supporting a role for the cytokines interleukin (IL)-6 and IL-1β in these effects (Pineda et al., 2013). The dependence of these effects on signaling *via* TLR3 was demonstrated by use of TLR3 gene deficient mice, albeit at adulthood, which show a reduced propensity to develop epileptic seizures after administration of the proconvulsant pilocarpine (Gross et al., 2017).

Several cytokines are known to have both acute and long-lasting effects on neuronal excitability, with IL-1β being the best characterized to date. Systemic or intracerebral administration of LPS or poly I:C to the mother rodent (or offspring; see the section *Infections in Postnatal Life*) provokes an acute elevation of pro-inflammatory cytokines *via* gene transcription of nuclear factor kappa-light-chain-enhancer of activated B cells (NF-κB) in neurons and glia of the mother and offspring (Pineda et al., 2013). By triggering a systemic immune response in the mother, gestational infections mimicked by LPS or poly I:C appear to also comprise the fetal placental barrier, allowing entry of maternally derived cytokines and other molecules (e.g. glucocorticoids) into the fetal circulation, where they can influence the developing fetal brain (Meyer, 2014). The extent to which maternally derived factors cross the placenta—a process known as “vertical transfer”—is incompletely understood, but appears to be cytokine-specific, and may be *via* either direct or passive transport (Zaretsky et al., 2004; Gilmore et al., 2005; Dahlgren et al., 2006). Regardless, abundant studies have demonstrated that a wide range of cytokines are increased in the fetal brain within hours after MIA in pregnant rodents, including IL-1β, tumor necrosis factor alpha (TNFα), and IL-6 (Solek et al., 2018). Activated microglia are likely to be a major source of inflammatory mediators in this context (Perry and Holmes, 2014). These cytokines can then act both directly and indirectly to modulate neuronal excitability and neurotransmission—for example, by altering the subunit composition of glutamatergic and gamma-aminobutyric acid (GABA)-ergic receptors (Vezzani et al., 2011a; Vezzani et al., 2011b; Vezzani and Viviani, 2015).

In the poly I:C MIA model, antibody blocking experiments were used to demonstrate that both IL-1β and IL-6 are required for an increased propensity for recurrent seizures (Pineda et al., 2013). Further evidence that IL-1β has a causative or modulatory role in network excitability stems from studies demonstrating anticonvulsive effects when IL-1β levels are reduced; either by intracerebral injection of an IL-1 receptor antagonist, transgenic overexpression of the receptor antagonist, or inhibition of interleukin-converting enzyme (Ravizza et al., 2008; Vezzani et al., 2011b).

## Maternal and Environmental Stress Throughout Development

Environmental stress during pregnancy, and early postnatal life, may promote the development of epilepsy during later life. Increasing evidence suggests that early life stress is an important modulator of limbic epilepsy, likely *via* effects on endocrine function, neuroplasticity, neurotransmission, and cellular electrophysiology (Koe et al., 2009). MIA and other forms of maternal stress, such as separation of dam from pups or early handling of pups, may also be considered as an early life stressor which aggravates epileptogenesis in both status epilepticus (SE) and kindling models (Salzberg et al., 2007). Additionally, stress resulting from transport of dams during pregnancy, as well as maternal behaviors and early postnatal malnutrition, have all been demonstrated to promote susceptibility to KA or amygdala kindling-induced seizures of the offspring during later life (Salzberg et al., 2007; Moriyama et al., 2013; Simao et al., 2016).

Maternal glucocorticoids as a consequence of a hyperactive hypothalamic–pituitary–adrenocortical (HPA) axis have been proposed to be one of the key mechanisms by which maternal stress mediates network reorganization and epileptogenesis in the developing offspring (Koe et al., 2009; Kumar et al., 2011; Jones et al., 2014; Wulsin et al., 2016). Another mechanism is *via* disruption of the GABA switch, a developmentally regulated functional change in GABA signaling from excitatory to inhibitory that occurs in the first 1–2 postnatal weeks (Ben-Ari, 2002). Neonatal stress from maternal separation has been found to delay the timing of the GABA switch in the mouse hippocampus, with consequences for behavior at adolescence (Furukawa et al., 2017). This study suggests that early life insults can disrupt or modify this essential step in GABAergic maturation and the resulting neuronal excitation/inhibition balance—which could have consequences for seizure propensity also. Indeed, the poly I:C MIA model has also been shown to delay the GABA switch in mice, resulting in hyperexcitability of neuronal networks and higher susceptibility to seizures at adulthood (Corradini et al., 2018).

Importantly, alterations in the stress response—resulting in elevated stress hormone levels—have been shown to promote chronic priming and activation of microglia, which then generate increased cytokines IL-1β, IL-6, and TNFα in response to a secondary immune challenge (Frank et al., 2014). This mechanism by which stress promotes increased neuroinflammation may thereby contribute to epileptogenesis in adulthood (Salzberg et al., 2007).

## Perinatal Insults

Hypoxic–ischemic injury (HI) is a significant cause of brain damage in newborn infants, and is associated with a high incidence of neurodevelopmental disabilities. Neonatal hypoxic–ischemic encephalopathy, defined as a syndrome of disturbed neurological function during the first days of life, often occurs following moderate to severe HI, and is the most common cause of neonatal seizures (Volpe, 1989; Zupanc, 2004). These acute seizures likely result from excitotoxic neuronal damage and cell death, following compromised oxygen and glucose supply to the developing brain. Epilepsy is reported in 9–33% of term infants with neonatal HI (Glass et al., 2011), with injury to the motor cortex, hippocampus, and occipital lobe being identified as risk factors for epilepsy in this context (Xu et al., 2019). Ischemic stroke is another common disorder affecting approximately one in every 4,000 live births, which is associated with both acute seizures and the subsequent development of epilepsy (Lynch and Nelson, 2001).

Animal models of perinatal hypoxia, HI, or stroke have suggested that the propensity for both acute seizures and epileptogenesis after injury is age-dependent (Jensen et al., 1998). Using the well-established Rice–Vannucci model of perinatal HI to p7 pups (Rice et al., 1981), acute seizures are associated with the extent of brain damage (Bjorkman et al., 2010), and spontaneous recurrent seizures have been reported in a subset of animals (Williams et al., 2004; Williams and Dudek, 2007). In another stroke model, involving induction of a photothrombotic lesion in the sensorimotor cortex of p7 rodents, seizure vulnerability was evaluated in response to the GABA_A_ receptor antagonist and pro-convulsive agent pentylenetetrazol (PTZ), an agent widely used to assess brain excitability (Klioueva et al., 2001). From electroencephalogram (EEG) analysis performed at 5 and 18 days post-injury (p12 and p25), early life stroke was found to result in an exacerbated response to PTZ, with a higher proportion of animals exhibiting clonic seizures, as well as longer response duration (Brima et al., 2013).

Hypoxia alone has also been shown to induce spontaneous tonic–clonic seizures in rodents, when induced at p10–12, but not in older (p15–60) or younger (p5) rats (Jensen et al., 1991; Jensen et al., 1992). These animals also displayed increased susceptibility to convulsant-induced seizures at adulthood, while hippocampal slices collected postmortem demonstrated chronic changes in CA1 hippocampal network excitability. Of note, these abnormalities were evident in the absence of overt histopathological damage or chronic neurobehavioral deficits (Jensen et al., 1991). In subsequent studies, hypoxic injury and HI at p7 have been shown to result in an increased vulnerability to KA challenge at 6 weeks post-injury (Rodriguez-Alvarez et al., 2015), as well as spontaneous epileptiform discharges and recurrent motor seizures by 2–12 months of age—but typically only in a subset of injured animals with cerebral cystic infarcts (Kadam et al., 2010; Peng et al., 2015). The frequency and severity of spontaneous behavioral and electrographic seizures increases over time, highlighting the progressive nature of epileptogenesis.

The contribution of inflammation to development of epilepsy in this context was recently demonstrated, by use of a novel therapeutic drug Vitexin. This anti-inflammatory botanical flavonoid was found to reduce cytokine release, neutrophil infiltration, and blood–brain barrier (BBB) permeability alongside a reduction in epilepsy susceptibility after HI in neonatal rats (Luo et al., 2018). In the clinic, in a small cohort study of patients with neonatal HI, elevated IL-6, TNFα, and IL-1β were found to be associated with the subsequent onset of epilepsy (Numis et al., 2019), suggesting that these cytokines may hold value as predictive biomarkers of later life epilepsy risk.

## Postnatal Insults

### Hyperthermia-Induced Febrile Seizures

Febrile seizures (FS), typically provoked by fever, are common during infancy and early childhood, affecting approximately 3–5% of children between 6 months and 5 years of age (Berg and Shinnar, 1996; Jensen and Baram, 2000). When recurrent or prolonged (approximately one-third of FS), these complex seizure events can lead to sustained modification and dysfunction of hippocampal neurons, which is proposed to underlie a heightened risk of subsequent epileptogenesis and neurocognitive dysfunction during later life (Dube et al., 2007; Huang and Chang, 2009). Epidemiological studies have linked prolonged FS during childhood with the development of TLE (Chen et al., 1999; Saltik et al., 2003; Fukuda et al., 2015), although whether this relationship is indeed causal, and the underlying mechanisms, remains unclear. Susceptibility to the convulsant effects of hyperthermia decreases with age in both humans and rodents, such that investigation into this phenomenon may provide insight into the mechanisms that govern this developmentally specific vulnerability during early life (Jensen and Baram, 2000).

Experimentally, rodent models of hyperthermia-induced FS typically utilize p10–14 animals (Heida et al., 2004; Heida and Pittman, 2005), consistent with the time period thought to represent the neurodevelopmental transition during the first 2 weeks of life in the human (Gottlieb et al., 1977; Semple et al., 2013). This model results in increased seizure susceptibility by adulthood, evident as a reduction in seizure threshold and increased susceptibility to seizure-induced cell death after a KA second-hit (Dube et al., 2000; Van Gassen et al., 2008). Approximately 40% of these animals develop spontaneous TLE alongside neuropathology in the cortex and hippocampus. Several factors have been proposed as mechanisms of epileptogenesis in this context, including the effects of altered brain temperature, changes in the endocannabinoid system, altered GABA_A_ subunit composition, and inflammation (Feng and Chen, 2016). In terms of the inflammatory response, the release and subsequent actions of IL-1β have also been strongly implicated (Feng and Chen, 2016). In patient populations, specific IL-1β polymorphisms have been associated with sporadic development of FS (Kira et al., 2005). Supporting this, administration of IL-1β after an induced FS in juvenile rats leads to a significant increase in seizure incidence compared to saline-treated controls (Fukuda et al., 2015), while IL-1β alone can mimic the effects of FS on adult seizure susceptibility (Feng et al., 2016). In another study, only rats in which IL-1β was elevated chronically went on to develop spontaneous limbic seizures after FS (Dube et al., 2010). In contrast, administration of the IL-1R antagonist is anticonvulsive (Heida and Pittman, 2005), while IL-1R–deficient mice are resistant to FS, independent of the genetic background strain (C57Bl or 129/Sv) (Dube et al., 2005). This mechanism holds strong promise for clinical translation, with two case reports demonstrating that treatment with the IL-1R antagonist reduced seizure burden and relapse in children with febrile infection-related epilepsy syndrome (Kenney-Jung et al., 2016; Dilena et al., 2019).

### Infections in Postnatal Life

Epidemiological evidence has demonstrated that CNS and systemic infections are another major cause of acquired epilepsy (Annegers et al., 1988; Marks et al., 1992). Indeed, an episode of viral encephalitis resulting from, for example, herpes simplex or cytomegalovirus, has been reported to increase the risk of subsequent unprovoked seizures by 16-fold (Annegers et al., 1988). These seizures are also associated with concurrent neurological consequences, and the increased risk for both epilepsy and neurobehavioral complications may persist even after the infection has resolved for at least 20 years (Annegers et al., 1988; Raschilas et al., 2002; Chen et al., 2006). In the juvenile rodent, poly I:C and LPS have been extensively utilized to model infection-like immune challenges during early postnatal life, and examine the effect of such insults on brain excitability.

#### Postnatal Poly I:C

In addition to use in the MIA model of a prenatal immune challenge, poly I:C is regularly employed to investigate infection-like immune challenges during early postnatal life. When injected directly into the rat hippocampus at p13–14, poly I:C induces fever and a local increase in IL-1β levels (Galic et al., 2009). Poly I:C facilitates electrical kindling epileptogenesis, as evident by an increased number of observed limbic seizures (Dupuis et al., 2016). Animals administered poly I:C at p13 demonstrated a faster seizure onset and prolonged kindled state compared to when the immune challenge was induced at adulthood (p74), again highlighting the age-dependent vulnerability of the early postnatal brain to hyperexcitability (Dupuis et al., 2016). Although microglia were hypothesized to play a role in these observations, administration of the tetracycline antibiotic minocycline, which has reported microglial suppressive effects (as well as other effects), prior to kindling, failed to reverse the pro-epileptogenic effects of poly I:C (Dupuis et al., 2016). In another study, animals exposed to poly I:C at p14 were found to be more susceptible to lithium-pilocarpine and PTZ-induced seizures at adulthood, and exhibited memory deficits in a fear conditioning paradigm (Galic et al., 2009). These chronic changes were coincidental with persistently altered levels of glutamate receptor subunits messenger ribonucleic acid (mRNA) expression, which were able to be suppressed by neonatal systemic minocycline, implicating a role for microglial activation as an underlying mechanism (Galic et al., 2009). Increased seizure susceptibility observed in adult rodents following poly I:C administration to young pups, similar to in the MIA context, is understood to depend at least in part upon early life activation of IL-1β signaling (Galic et al., 2009). Together, these studies demonstrate that poly I:C exposure is pro-epileptic in the early postnatal brain, similar to the prenatal brain, although the precise mechanisms (and whether these are age-dependent) remain incomplete.

#### Postnatal LPS

Perhaps the most common experimental model of postnatal infection involves administration of LPS, typically to the periphery (intraperitoneal) to p10–16 rodent pups (Galic et al., 2008; Auvin et al., 2010a). Depending upon the dose, LPS induces a transient inflammatory response within the first 12–24 h post-injection which largely resolves thereafter. Of note, a persistent increase in seizure susceptibility is evident following low dose LPS at p7–14—compared to when LPS was administered earlier (p1) or later (p20) (Galic et al., 2008). This time period coincides with the developmental peak in synaptogenesis and synaptic pruning, yielding considerable changes in neuronal circuitry which likely underlies this critical window of vulnerability. This paradigm has since been used to demonstrate that LPS exposure increases susceptibility to KA-induced seizures at p35, alongside impairments in long-term potentiation and exacerbated hippocampal neurodegeneration (Chen et al., 2013), and pilocarpine-induced seizures at 2 months of age (Setkowicz et al., 2017). In another study, KA was administered simultaneously with LPS to p14 pups, revealing long-lasting molecular changes alongside increased seizure excitability by adulthood. This was enhanced further in the ~50% subset of LPS + KA–treated animals that exhibited an overt behavioral seizure response FS compared to those that did not, as evidenced by increased *in vitro* excitability as well as modified N-methyl-D-aspartate (NMDA) and GABA_A_ receptor subunit protein expression in the hippocampus (Reid et al., 2013).

Similarly, LPS has been shown to potentiate fever-induced FS at p14 in both rats and mice, at least acutely (Eun et al., 2015). Administered peripherally 2 h prior to hyperthermia-induced seizure onset, LPS promoted susceptibility of animals to seizures, alongside enhanced pro-inflammatory cytokine production and microglial activation. These findings suggest that peripheral inflammation works synergistically with hyperthermia to potentiate seizures and exacerbate the resultant immune response. Whether this combinatorial challenge also predicts heightened vulnerability to chronic epilepsy has not yet been examined.

Several studies have noted that the inflammatory challenge during early life is typically transient; for example, LPS-induced acute inflammation rarely lasts more than 24 h following injection. Thus significant changes in the reactivity to seizures observed in adulthood do not directly result from inflammation *per se,* but rather indirectly from the long-term effects of the acute inflammatory response on the immature brain, and its subsequent developmental trajectory (Kosonowska et al., 2015; Janeczko et al., 2018).

LPS administration triggers the abundant release of IL-1β and TNFα, which can act on receptors such as IL-1R on the hippocampal dentate gyrus to facilitate enhanced epileptiform activity (Gao et al., 2014). A central role for these cytokines has been demonstrated in the context of epileptogenesis following LPS in the p14 rat, which can be partially prevented by administration of either IL-1R antagonism or an anti-TNFα antibody (Galic et al., 2008; Auvin et al., 2010b). LPS-induced seizure susceptibility was also recently shown to involve TLR4 activation, signaling *via* extracellular signal–regulated kinases 1 and 2 (Erk1/2), in a manner dependent on myeloid differentiation primary response 88 (MyD88) (Shen et al., 2016). Constitutive activation of Erk1/2 in astrocytes alone was sufficient to enhance excitatory synaptogenesis, while deleting MyD88 or suppressing Erk1/2 in astrocytes was able to ameliorate seizure sensitivity, providing direct evidence for a developmental role of astrocytes in predisposing towards epileptogenesis (Shen et al., 2016).

**Figure 2:**
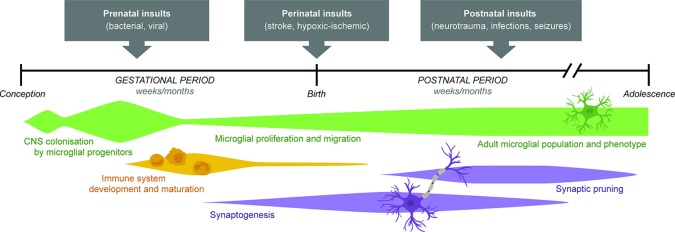
Schematic timeline illustrating key neurodevelopmental processes ongoing through gestational and postnatal periods in the mammalian brain. A wide range of prenatal, perinatal, and postnatal insults influence the developing brain both acutely but also chronically, driving an increased propensity for neuronal hyperexcitability and seizure susceptibility during later life. Age-dependent vulnerability to these chronic consequences is thought to be determined, at least in part, by the state of microglial development (changes in number, phenotype, and activity), as well as maturation of neuronal circuits (a product of synaptogenesis and synaptic pruning over time). Adapted from Semple et al. (2013) and Lenz and Nelson (2018).

As noted earlier, a particular window of enhanced vulnerability to seizures has been identified in rodent models during the second week of postnatal life, coincidental with significant synaptogenesis and synaptic pruning (Galic et al., 2008). As microglia are known regulators of synaptic remodeling (Tremblay et al., 2011), and peak in cell density at around p14–28 (Kim et al., 2015), microglia may also govern this age-dependent susceptibility (**Figure 2**). Heightened reactivity due to normal developmental changes render microglia particularly poised to mount an exaggerated inflammatory response to early life seizures or other immune challenges that are encountered at this time; and this over-reactive immunity may exacerbate acute neuronal injury thereby contributing to long-term epileptogenic effects (Tremblay et al., 2011; Kim et al., 2015).

### Postnatal Status Epilepticus

SE is defined as a condition in which abnormally prolonged seizures occur, which may have long-term consequences including neuronal loss and altered neuronal networks (Trinka et al., 2015). In experimental animals, a transient episode of SE can “convert” a previously normal brain into an epileptic one, providing a model in which to explore mechanisms of epileptogenesis (Lothman and Bertram, 1993). Of note, vulnerability to KA appears to be age-specific, with younger animals (p5–15) exhibiting more severe SE with a shorter latency, and higher mortality, compared to older animals (p20–60) (Holmes and Thompson, 1988; Stafstrom et al., 1992).

Microglia, which mediate a significant proportion of the innate immune capacity of the CNS, are critical for immune surveillance in the steady state, as well as the response to injury and disease (Disabato et al., 2017). Chronic microglial activation is a common component to a wide range of neurodegenerative conditions including multiple sclerosis, Alzheimer's disease, and traumatic brain injury (TBI), likely contributing to neuronal dysfunction and cell loss to facilitate disease progression. KA-induced SE triggers a time-dependent microglial activation response including the release of pro-inflammatory cytokines TNFα and IL-1β (Wyatt-Johnson et al., 2017), which appears to precede the appearance of neuronal damage (Rizzi et al., 2003; Ravizza et al., 2005). In this context, IL-1 signaling has again been implicated, with experiments in which IL-1β was administered prior to KA reported an increase in the time spent in seizures *via* an NMDA receptor–dependent mechanism (Vezzani et al., 1999).

In some instances, in response to an immune challenge, microglia are induced to a “primed” state—not activated *per se*, but in an intermediate phenotype which renders them able to respond more rapidly when subsequently activated, including the production of greater quantities of pro-inflammatory cytokines compared to normally activated, non-primed (or quiescent) microglia (Sparkman and Johnson, 2008). This appears to be the case after early life KA-induced seizures in p15 rats, followed by a second-hit exposure of KA at p45. Animals that were exposed to both KA doses had greater microglial activation, associated with elevated pro-inflammatory cytokine levels and increased susceptibility to seizures compared to saline-control animals that received the KA only at p45 (no prior exposure) (Somera-Molina et al., 2009). Treatment with Minozac, a small novel therapeutic compound that inhibits pro-inflammatory cytokine production, attenuated these effects. These results implicate cytokines produced by activated microglia as one mechanism by which early life seizures contribute to increased vulnerability to neurological insults in adulthood (Somera-Molina et al., 2009). Similarly, administration of minocycline following KA at p25 in mice has been shown to reduce vulnerability to a second SE event at p39, likely to be attributed to the suppression of microglial activation—providing further evidence that early life insults such as seizures act to prime microglia for a subsequent immune challenge (Abraham et al., 2012).

### Early Life Neurotrauma

TBI during early childhood is another well-known cause of epilepsy. This post-traumatic epilepsy (PTE) has a reported incidence of up to 35% after severe TBI (Annegers et al., 1980; Ates et al., 2006; Arndt et al., 2013). While several well-characterized experimental models have been utilized in adult rodents to explore PTE after TBI to the mature brain (D'ambrosio et al., 2004; Bolkvadze and Pitkanen, 2012; Kelly et al., 2015; Ostergard et al., 2016), there has been a lack of age-appropriate models to consider the complex interaction between ongoing brain development and epileptogenesis that occurs after a TBI during early childhood.

An established model of experimental TBI to the p21 mouse, utilizing the controlled cortical impact model of unilateral injury to the parietal lobe, results in progressive neuropathology and chronic neurobehavioral and neurocognitive dysfunction consistent with what is commonly observed in toddler-aged children after TBI (Tong et al., 2002; Pullela et al., 2006). This model has recently been demonstrated to also reproduce many of the features characteristic of PTE in humans, including histopathological evidence of circuitry reorganization, interneuron loss, and hippocampal gliosis (Semple et al., 2017). Brain-injured mice exhibit both an increased vulnerability to PTZ-evoked seizures, evident as early as 2 weeks post-TBI, suggesting that epileptogenesis is underway at this time creating an environmental primed for the development of PTE. A proportion of TBI mice were reported to develop at least one spontaneous seizure within a 7-day video-EEG recording period by 4–6 months post-injury—from 15% after a moderate injury severity up to over 90% incidence after a severe injury involving considerable hippocampal pathology (Semple et al., 2017; Webster et al., 2019).

Although the mechanisms underlying PTE remain unclear, several lines of evidence point towards a prominent role of cytokine signaling, particularly *via* IL-1 (Webster et al., 2017). Experimentally, administration of the IL-1R antagonist attenuates both sub-acute and chronic susceptibility to PTZ-induced seizures after pediatric TBI in the mouse (Semple et al., 2017). Genetic data from patient populations has also implicated specific IL-1β polymorphisms with the risk of PTE after a TBI (Diamond et al., 2015).

This latter point raises and somewhat addresses an important question—why do some individuals, a minority, respond to an early life insult with epilepsy, while others who sustain a similar insult do not? Our understanding of how environmental factors (such as an early life insult) interact with genetics to promote epileptogenesis remains in its infancy. However, there is increasing evidence that genetic predisposition to epilepsy will increase an individuals' likelihood of developing late-onset seizures after an acquired insult. For example, a higher risk of post-stroke epilepsy was recently reported in individuals with a family history of epilepsy compared to those without a family history, even when adjusted for stroke injury severity (Eriksson et al., 2019). Similarly, in a population-based cohort study of more than 1.6 million Danish adults and children, a family history of epilepsy was associated with an approximately 10-fold higher risk of developing late-onset seizures following a severe brain injury (Christensen et al., 2009). Limited studies to date have specifically probed for gene associations with acquired epilepsy risk, as recently reviewed (Leung et al., 2019). Consistent with the abovementioned evidence on IL-1β polymorphisms and PTE risk, a meta-analysis found that specific alleles of both IL-1β and IL-1α have also been associated with risk of epilepsy after FS (Saghazadeh et al., 2014). Further investigation into other genes involved in the inflammatory response, and in a range of patient populations, are needed to determine the extent to which genetic variance contributes to an individual's risk of epilepsy after an early life insult.

## Inflammatory Preconditioning—Protection *Against* Epilepsy?

Contrary to the above-described literature, there is also preclinical evidence that an early life insult inducing a modest inflammatory response can alternatively attenuate the response to a second-hit insult. This phenomenon, termed “preconditioning,” occurs when the brain develops resistance to injury after exposure to a low dose, typically subthreshold stimuli, such as brief ischemia, hypoxia, or low dose endotoxin. Preconditioning in the context of brain insults has been well documented in adult animals, but less so in the immature brain. Even fewer studies have considered the effect on neuronal excitability and seizure vulnerability.

Administration of a low dose of LPS (typically in the 0.05–1.0 mg/kg range) (Hickey et al., 2011) is one of the best-characterized approaches to yield neuroprotection *via* preconditioning. Acting *via* TLR4, LPS is thought to reprogram the intracellular response to a subsequent insult, resulting in broad neuroprotection *via* activation of anti-inflammatory factors, alongside the downregulation of NF-κB (Hickey et al., 2011; Wang et al., 2015; Amini et al., 2018). One study compared young rats exposed to systemic low dose LPS at p6 and p30, followed by pilocarpine injection at 2 months of age, and reported that LPS at p30 only resulted in a reduction in acute seizures alongside ameliorated seizure-induced changes in microglial morphology (Kosonowska et al., 2015). Anti-ictogenic effects have also been reported following TLR3 activation in adult mice, whereby intraventricular poly I:C administered 6 h prior to a KA challenge was found to prevent the anticipated increase in hippocampal excitability (Kostoula et al., 2019). This effect was mimicked by administration of the cytokine interferon gamma, suggesting that activation of downstream signaling *via* interferon regulatory factor 3 is involved (Kostoula et al., 2019). Of note, poly I:C administered at 15 min, 1 h, or 24 h prior to KA failed to elicit anti-ictogenic effects, suggesting that the timing is crucial and likely involves the activation of transcriptional rather than posttranslational mechanisms to influence neuronal excitability.

A role for astrocytes in modulating the relative levels of pro- versus anti-inflammatory mediators has been reported as a biological mechanism associated with this phenomenon, as well as microglial priming (Kosonowska et al., 2015). In addition to modulation of the inflammatory response, several other cellular and molecular mechanisms have been proposed to underlie preconditioning neuroprotection, for example, changes in calcium binding, transcriptional regulation, apoptosis, growth, and development processes (Friedman et al., 2013; Friedman and Hu, 2014). The apparent paradox regarding why an early life insult may induce either seizure susceptibility or resistance (alongside neuroprotection) is poorly understood. However, it may be that TLR3 and TLR4 have dual roles whereby activation of alternative pathways in different cell types yields differential consequences. It is clear that the preconditioning phenomenon is both age and dose dependent (Hickey et al., 2011). Further, the time interval between the first and second insult is likely to be an important determinant. For example, although seizure susceptibility was not examined, one study found that low dose LPS administered at 48 h before HI in p7 rats was neuroprotective, whereas administration earlier at 72 h before HI instead increased the extent of brain damage (Hickey et al., 2011).

## Conclusions

In this review, we have summarized and critically discussed the most common known causes of acquired epilepsy following injury or insult during early life, from maternal infection exposure through to TBI during young childhood. We have excluded from our discussion some other causes of acquired epilepsy, such as brain tumors (Weisman et al., 2018) or malformation of cortical development (e.g. focal cortical dysplasia) (Crino, 2015), based on the observation that these factors often persist throughout a patients' life span—rather than being a transient early life insult that resolves with time, in the face of persistent seizure susceptibility, as we have focused on in this review.

All of the described early life insults induce activation of the innate immune response, eliciting reactivity of glial cells, release of pro-inflammatory mediators, and neuronal or network changes in favor of a more excitable CNS microenvironment, which appears to facilitate the process of epileptogenesis and subsequent emergence of spontaneous recurrent seizures (or increased vulnerability to evoked seizures) at a later time (**Figure 1**). To date, the evidence supports that early life challenges act as risk factors for epilepsy, but do not necessarily cause epilepsy *per se*; indeed, genetic predisposition, environmental factors, and interactions between all of these variables are likely to determine an individual's risk status (Koe et al., 2009). Experimental models have been invaluable to determine particular developmental windows of increased vulnerability to insult, whereby the brain is rendered immunologically primed and more reactive to a second-hit insult should one occur (**Figure 2**). Transient cytokine release (including IL-1β, TNFβ, and IL-6; see **Table 1**), microglial priming, and astrocyte reactivity are all mechanisms by which early life immune challenges can yield long-lasting effects on seizure threshold. Several other cytokines, chemokines, and damage-associated molecular patterns including IL-6 and high mobility group box protein-1 have also been implicated in seizure ictogenesis and epileptogenesis, as reviewed extensively elsewhere (Vezzani et al., 2008; Vezzani and Viviani, 2015; Webster et al., 2017; Vezzani et al., 2019). However, few studies to date have studied these mediators in the context of how early life immune challenges promote later onset epilepsy.

**Table 1:** Key inflammatory mediators implicated in epileptogenesis after early life insults: experimental evidence.

Mediator	Insult	Model	Species/Age	Effect and Potential Mechanisms	Reference(s)
**IL-1β**	Bacterial infection (MIA; postnatal infections)	Systemic or intracerebral LPS administration	Rat, p14 Rat, g15–16	After p14 LPS, increased IL-1β production in response to KA at adulthood After LPS + sub-convulsive KA, i.c.v. IL-1β increased the proportion of animals with seizures, while IL-1R antagonist was anticonvulsive IL-1R modifications associated with hyperexcitability, upregulation of NF-κB, and altered GABAergic subunit expression	(Heida and Pittman, 2005; Vezzani et al., 2008; Chen et al., 2013)
	Preterm HI injury	In utero HI + LPS administration	Rat, g18	Increased placental IL-1β acutely and sub-acutely associated with fetal neuroinflammation and neuronal injury	(Maxwell et al., 2015)
	Viral infection (MIA; postnatal inoculation)	Systemic or intracerebral poly I:C administration	Mouse, g12–16 Rat, p13–14	Chronic epilepsy phenotype in offspring prevented by antibodies to IL-1β and IL-6 (when combined) Increased IL-1β associated with kindling epileptogenesis	(Galic et al., 2009; Pineda et al., 2013; Dupuis et al., 2016)
	Infantile FS	Hyperthermia induction ± intranasal IL-1β	Rat, p10–12 Mice, p14–15	Addition of intranasal IL-1β increased seizures following KA at p70–73, associated with hippocampal cell loss in CA3 region Hippocampal IL-1β levels only in rats that developed late-onset seizures Exogenous IL-1β exacerbates FS, while IL-1R–deficient mice show resistance to FS mRNA levels of IL-1R correlates with epilepsy-predictive MRI signal changes	(Dube et al., 2005; Dube et al., 2010; Fukuda et al., 2014; Fukuda et al., 2015; Patterson et al., 2015)
	Status epilepticus	KA kindling; lithium-pilocarpine	Rat, p9–15	Upregulated IL-1β and IL-1R acutely and chronically (to 8 weeks), associated with glial activation	(Holmes and Thompson, 1988; Rizzi et al., 2003; Omran et al., 2012)
	Trauma	Controlled cortical impact	Mouse, p21	Upregulation of IL-1β and IL-1R acutely; prevention of chronic seizure susceptibility by treatment with IL-1R antagonist	(Semple et al., 2017)
**IL-6**	Bacterial infection	Systemic or intracerebral LPS administration	Mouse, p10–14 Rat, p6	Increased IL-6 acutely post-LPS associated with chronically activated microglia	(Kosonowska et al., 2015)
	Infantile FS	Hyperthermia induction ± IL-6 administration	Rat, p23–28	IL-6 dose-dependently reduced hyperthermia-induced seizures—anticonvulsive effect	(Fukuda et al., 2007)
	Viral infection (MIA)	Systemic or intracerebral poly I:C administration	Mouse, g12–16	Chronic epilepsy phenotype in offspring prevented by antibodies to IL-1β and IL-6 (when combined)	(Pineda et al., 2013)
	Prenatal immune challenge (IL-6)	Systemic IL-6 administration	Mouse, g12–16	In combination with IL-1β, increased propensity to hippocampal kindling, associated with social deficits	(Washington et al., 2015)
	Status epilepticus	KA kindling	Rat, p9–21	Upregulated IL-6 acutely after KA, associated with glial activation	(Rizzi et al., 2003)
**TNFα**	Bacterial infection	Systemic or intracerebral LPS administration	Rat, p6–7, p14	Increased TNFα acutely post-LPS, and in response to KA at adulthood Response to lithium-pilocarpine, KA, and pentylenetetrazol at adulthood was mimicked by i.c.v. recombinant TNFα and blocked by an anti-TNFα antibody	(Galic et al., 2008; Chen et al., 2013; Kosonowska et al., 2015)
	Bacterial infection (meningitis)	*S. pneumoniae* inoculation	Rat, p11	TNFα-converting enzyme attenuates incidence of seizures and exerts neuroprotection	(Meli et al., 2004)
	Preterm HI	In utero HI + LPS administration	Rat, g18	Increased placental IL-1β acutely and sub-acutely associated with fetal neuroinflammation and neuronal injury	(Maxwell et al., 2015)
	Status epilepticus	KA kindling; lithium-pilocarpine	Rat, p9–21 Rat, p25	Upregulated TNFα acutely after KA associated with glial activation Upregulated TNFα and chronically in pilocarpine model of TLE, associated with astrocyte activation	(Rizzi et al., 2003; Ashhab et al., 2013)

BBB, blood–brain barrier; DAMP, damage-associated molecular pattern; g, gestational day; GABA, gamma-aminobutyric acid; FS, febrile seizure; HCN, hyperpolarization-activated cyclic nucleotide-gated channel; HI, hypoxic–ischemic injury; HMGB1, high-mobility group box protein-1; HPA, hypothalamic–pituitary axis; i.c.v., intracerebroventricular; IL, interleukin; IL-1R, interleukin-1 receptor; KA, kainic acid; LPS, lipopolysaccharide; mRNA, messenger ribonucleic acid; MIA, maternal immune activation; NF-κB, nuclear factor kappa-light-chain-enhancer of activated B cells; NMDA, N-methyl-D-aspartate; NMDAR, N-methyl-D-aspartate receptor; p, postnatal day; poly I:C, polyinosinic:polycytidylic acid; PTZ, pentylenetetrazol; S. pneumoniae, Streptococcus pneumoniae; TBI, traumatic brain injury; TLE, temporal lobe epilepsy; TLR, toll-like receptor; TNFα, tumor necrosis factor alpha.

Future studies to identify and characterize the key factors mediating the chronic consequences of such insults may allow for the development of predictive tests to more readily identify those individuals at greatest risk. Further, novel immune-based therapies may provide therapeutic benefit by aborting the epileptogenesis process prior to the onset of spontaneous recurrent seizures, or even mitigating its severity after the onset of epilepsy (i.e. disease modifying).

## Author Contributions

BS conceptualized and drafted the manuscript. LD and TO'B provided critical revision and intellectual input, and LD generated the figure. BS and LD incorporated reviewer feedback to revise the manuscript for resubmission. All authors approve the manuscript and are accountable for its content.

## Funding

The authors acknowledge funding support from the National Health and Medical Research Council of Australia (NHMRC #1122456, 1141347 and 1176426) and Monash University, Australia. 

## Conflict of Interest

The authors declare that the research was conducted in the absence of any commercial or financial relationships that could be construed as a potential conflict of interest.

The reviewer AV declared a past co-authorship with one of the authors, TO'B, to the handling editor.
